# Recent Advances in the Production and Applications of Ellagic Acid and Its Derivatives. A Review

**DOI:** 10.3390/molecules25122745

**Published:** 2020-06-13

**Authors:** Dmitry D. Evtyugin, Sandra Magina, Dmitry V. Evtuguin

**Affiliations:** CICECO/Department of Chemistry, University of Aveiro, Campus Universitário de Santiago, 3810-193 Aveiro, Portugal; dmitry.evtyugin@ua.pt (D.D.E.); smagina@ua.pt (S.M.)

**Keywords:** ellagic acid, ellagitannins, urolithins, antioxidant properties, biological activity, bioavailability, production routes

## Abstract

Ellagitannins (ETs), characterized by their diversity and chemical complexity, belong to the class of hydrolysable tannins that, via hydrolysis under acidic or alkaline conditions, can yield ellagic acid (EA). They are mostly found as a part of extractives in angiosperms. As known antioxidants and chelators, EA and EA derivatives are drawing an increasing interest towards extensive technical and biomedical applications. The latter ones include possible antibacterial, antifungal, antiviral, anti-inflammatory, hepato- and cardioprotective, chemopreventive, neuroprotective, anti-diabetic, gastroprotective, antihyperlipidemic, and antidepressant-like activities, among others. EA’s synthesis and production challenges prompt further research on new methods and alternative sources. Conventional and prospective methods and raw materials for the production of EA and its derivatives are reviewed. Among the potential sources of EA, the residues and industrial streams of the pulp industry have been highlighted and considered as an alluring alternative in terms of commercial exploitation.

## 1. Introduction

**Figure 1:**
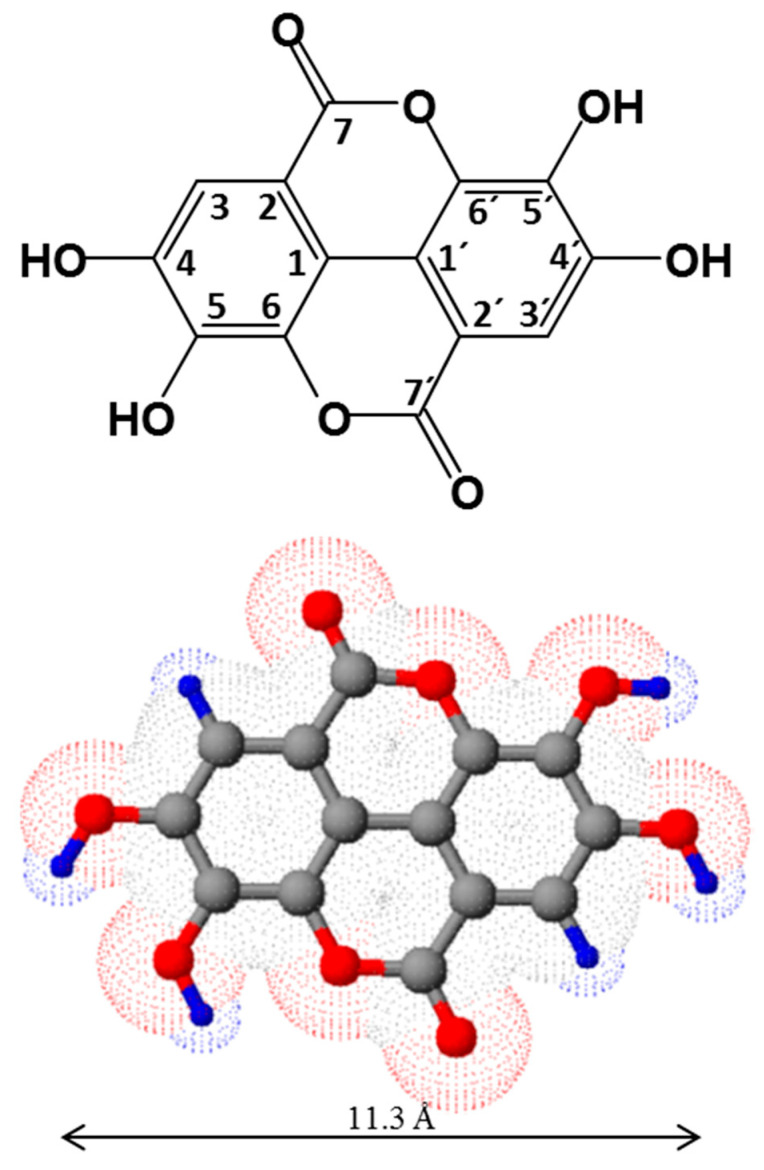
Chemical structure of ellagic acid.

Ellagic acid (EA) (Figure 1), belongs to the class of polyphenol extractives (tannins) widely spread among dicotyledons [1]. In plants, EA is predominately found ester-linked to sugars in the composition of hydrolysable tannins called ellagitannins (ETs). Among hydrolysable tannins, with more than a 1000 identified molecules, ETs form the largest group [2,3]. As other tannins, ETs are secondary metabolites of higher plants [2] and act as a part of the defense mechanism against microbial and animal attacks due to their astringent capacity and the ability to form complexes with proteins and polysaccharides [1]. During plant chemical processing, both under acidic or basic conditions, ester bonds of ETs are hydrolyzed, yielding a hexahydroxydiphenoyl (HHDP) group, which spontaneously lactonizes into the almost water-insoluble ellagic acid (EA).

Hydrolysable tannins have long been known for their use in leather tanning processes [1,4]. Nonetheless, today the growing interest in these compounds is mainly associated with the consumption and development of new products offering beneficial health effects linked to phenolic antioxidant properties [5]. Accordingly, owing to beneficial health effects against many oxidative-linked chronic diseases, including cancer and neurodegenerative diseases, EA has generated a noticeable scientific interest [6,7,8,9,10,11,12,13]. Beyond food and biomedical applications, EA and ET’s scientific relevance can be linked to advanced materials such as copolymers [14], chelating reagents [15], ion-exchange resins [16], and materials for electrochemical devices [17], among others. Meanwhile, due to the presence of ETs in the heartwood of most *Eucalyptus* species, these are hydrolyzed to EA, resulting in manufacturing problems for the pulp industry, such as excessive chemical consumption during pulping, dark coloring of pulps [18,19] and insoluble deposits (pitch) on the metal equipment surface [20]. At the same time, the pulp industry could be considered the largest producer of underutilized hydrolysable tannins, including EA/ETs [21]. This review aims to bring new perspectives to the scientific community on recent advances in the production and application of ellagic acid and its derivatives in food, biomedical and technical areas. Since many practical applications of EA are hampered by poor market availability and high prices, this review also intends to emphasize new challenges facing EA production not only from fruits, nuts or herbs, but also from industrial streams of wood processing industries.

## 2. The Chemistry of Ellagic Acid and Ellagitannins 

### 2.1. Structure and Physico-Chemical Properties of Ellagic Acid

Ellagic acid (EA), first noticed by Chevreul in the gallnut (noix de galle in french), was described in 1818 by Braconnot [22], who named the acid by reversing the word “galle” [23]. EA consists of a dimeric derivative of gallic acid with a molecular weight of 302.194 g/mol. According to IUPAC nomenclature, EA is identified as 2,3,7,8-tetrahydroxychromeno[5,4,3-cde]chromene-5,10-dione, though the most common designation in chemistry may be found based on diphenic acid classification (4,4′,5,5′,6,6′-hexahydroxydiphenic acid 2,6,2′,6′-dilactone). EA comprises four free OH groups and two acyloxy groups linked to a core of fused aromatic rings (Figure 1), keeping a near planar structure with molecular symmetry C*_2h_* and crystallizing in the monoclinic cell, space group *P*2_1_/c [24]. EA dihydrate forms triclinic crystals representing characteristics of the *P*1 space group [25]. Concomitants and eventual metal complexes explain the variety of different crystalline groups of EA isolated from natural sources [17].

The assignments to proton and carbon resonances in NMR spectra of EA are widely reported [17,26]. Electron-donating groups enhance EA’s electron density, bestowing EA to participate in hydrogen bonding and π–π interactions. Thus, such characteristics are directly related to EA’s diversity in terms of practical uses. The four phenolic and two lactone groups form the hydrophilic part, while two phenyl rings represent the hydrophobic part, hence EA exhibits amphiphilic character (Figure 1). Given its low polarity, EA is only sparingly soluble in aqueous media (9.7 µg/mL at 37 °C) [27]. Meanwhile, the solubility of EA is increased substantially in methanol (671 µg/mL at 37 °C) [27]. EA’s high solubility in pyridine has also been documented [17,27]. The most promising results for pharmaceutical use include *N*-methyl pyrrolidone (skin penetration enhancer for transdermal use), polyethylene glycol 400 (vehicle for parenteral dosage forms) and triethanolamine (salt formation in injectable and topical preparations) with small amounts of water [27].

The free radical scavenging activity of phenolics is influenced by the pH of the surrounding medium [28]. EA can be partially or fully ionized, suggesting that ions could also be involved in the antioxidant activity and underlining the importance of EA protolytic equilibria studies. All four phenolic groups can suffer deprotonation, which would suggest four p*K*_a_ values. However, due to symmetric phenolic substituents in EA, usually two p*K*_a_ values assigned to 4-/4′-OH and 5-/5′-OH are referred. Simić and co-workers [29] clearly detected two acidity constants p*K*_a1_ and p*K*_a2_ of 5.42 and 6.76, respectively, confirming the diprotic nature of EA. Therefore, three different regions were recognized, depending on different dominating species: unionized molecule (H_4_A), monoanion (H_3_A^−^), and dianion (H_2_A^2−^).

Free radical scavenging activity of EA relates to the phenolic H-atom transfer (HAT), single electron transfer followed by proton transfer (SET-PT), and sequential proton loss electron transfer (SPLET) mechanisms. By analyzing the energy requirements for bond dissociation enthalpy (BDE), adiabatic ionization potential (IP), O-H proton dissociation enthalpy (PDE), proton affinity (PA), and electron transfer enthalpy (ETE), it is possible to indicate which mechanism is thermodynamically favored and identify the active site for radical inactivation [30,31]. Thus, BDE characterizes the HAT mechanism; IP and PDE the SET-PT mechanism; finally, PA and ETE the SPLET mechanism. Marković and co-workers [30] calculated these parameters for ellagic acid and its phenoxide anions, bringing some insightful conclusions regarding the antiradical mechanism of EA.

### 2.2. Structure of Ellagitannins

**Figure 2:**
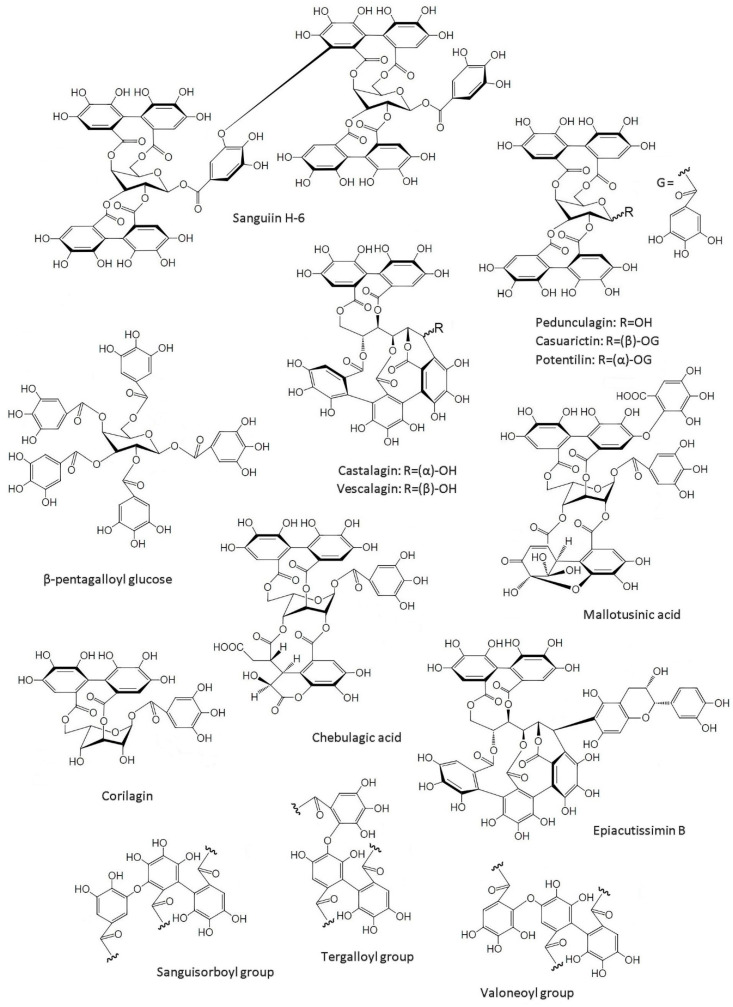
Example of some ellagitannin structures and their precursor, β-pentagalloyl glucose.

ET’s complexity and diversity are directly linked to their biosynthetic variability, and there are limitless possible structures as a result thereof. In fact, more than 1000 ETs have been identified to date. Some ET structures, characteristic groups and their precursor, β-pentagalloyl glucose, are depicted in Figure 2. ETs are formed via oxidative C-C coupling of at least two galloyl units of the β-pentagalloyl glucose (Figure 2), leading to an axially chiral HHDP unit [1]. Further steps can lead to the formation of a second HHDP group (e.g., Casuarictin, Figure 2) or to the cleavage of the formed HHDP or galloyl groups (e.g., Corilagin, Figure 2). Trimer and tetramer forms of the galloyl group can result from a further oxidative coupling. Such is the case of Castalagin and Vescalagin (Figure 2), which have a nonahydroxytriphenoyl (NHTP) group, also known as flavogallonyl. HHDP groups can also suffer further oxidation to form other units, such as dehydrohexahydroxydiphenoyl (DHHDP) (e.g., Mallotusinic acid in Figure 2) or chebuloyl (e.g., Chebulagic acid in Figure 2). C-O bonding of HHDP groups is another possibility, resulting in sanguisorboyl, tergalloyl and valoneoyl groups (Figure 2), among others [32]. Thus, via the oxidative C-O coupling between galloyl and hexahydroxydiphenoyl moieties, ET monomers can form dimers, trimers and tetramers with molecular weights up to several thousands of Da (e.g., Sanguiin H-6, a Casuarictin dimer, in Figure 2). The nature of the bonds between monomers, either biphenyl or diarylether, sets up a method for their classification [32]. Lastly, ETs can give rise to hybrid structures by joining with other classes of molecules: e.g., Epiacutissimin B (Figure 2), a flavano-ellagitannin, has epicatechin at the C-1 center of the open-chain glucose core [33].

It is certain that the pentagalloyl glucose oxidation pathway plays a central role in ellagitannins biosynthesis, but differing structural principles have been recognized for this class, which still leave many gaps, not only in the identification of enzymes catalyzing the synthesis of different linkage types, but also regarding some physiological aspects, such as seasonal variation of metabolite concentrations and enzyme activities [34]. Detailed postulations on ET’s biosynthesis fall outside of the scope of this review and can be found elsewhere [1,34]. Additionally, a detailed discussion on structural revisions of some ETs can be found in a recent review [3], reinforcing once again the complexity and structural diversity of this tannin class.

## 3. Sources of Ellagic Acid and Ellagitannins

ETs are known constituents of numerous species of economic importance [1]. They are abundant in berries of the family *Rosaceae* such as cloudberry, raspberry and strawberry. They seem to have most of their EA in the form of ETs, as the relative amount of free EA and its glycosides is rather low [35,36]. In general, the amount of EA/ET in fruits can range from 100 to 1500 mg·kg^−1^ and contributes substantially to the dietary intake [27]. Kakadu plum, with up to 140.2 g·kg^−1^ (dw) of EA, is probably the richest edible source [37,38]. Other important sources of ETs include walnuts [39], pecans [40], camu-camu fruits [41], pomegranates [42], and muscadine grapes [43]. The amounts of EA/ETs found in different fruits, nuts and woods are summarized in Table 1. Notably, pomegranate peel has been considered as a prominent source of raw material for industrial exploitation [44].

Many medicinal plants used for their antioxidant, anti-diarrheic and anti-microbial activities contain ETs. Some notable examples include Agrimoniin (*Agrimonia pilosa*), Camelliatannin A (*Camelia japonica*), Casuarictin (*Liquidambar formosana*), Chebulinic acid (*Terminalia chebula*), Cornussin A (*Cornus officinalis*), Gemin-A (*Geum japonicum*), Geraniin (*Geranium thunbergii*), Granatin B (*Punica granatum*), Mallotusinic acid (*Mallotus japonicas*), Oenothein B (*Oenothera erythrosepala*) and Rugosin (*Rosa rugosa*) [32]. Lastly, EA, methyl derivatives of EA, and glycosides of both, are the components of the tannin extractives of *Eucalyptus* species [45]. Therefore, EA is also present in agro-forest and industrial residues (e.g., in cork, tree bark and wood) [46]. In fact, Santos and co-workers [47] reported 512.8 mg·kg^−1^ (dw) of EA in Brazilian *E. grandis* and these values are in accordance with their previous findings. Moreover, using the capillary zone electrophoresis (CZE) analytical procedure, reliable determinations have been made of EA in *E. globulus* wood: 1100 ± 600 mg·kg^−1^ (dw) [48]. Usually, eucalypt bark contains 3–5 times higher EA/ETs than wood [49]. Besides *Eucalyptus*, EA/ETs are also widely present in *Quercus* [50], *Acacia* [51] and *Castanea* [52] species, among some other angiosperms [53]. It is noteworthy that the abundance of EA and ETs in wood and bark is comparable or even higher than in most agricultural sources (Table 1).

**Table 1:** Sources of EA and its content (mg·kg^−1^) in different fruits, nuts, seeds and woods *.

Source	Latin Name	Total ET/EA ^#^	Free EA	Ref.
**Fruits**				
Arctic bramble	*Rubus arcticus*	3900 (fw)	-	[36]
Blackberry	*Rubus ursinus*	1500 ± 140 (dw)	-	[54]
Camu-camu fruit:	*Myrciaria dubia*			[41]
Pulp powder		258.5 ± 4.3 (dw) *	56.0 ± 1.1 (dw)	
Flour		5656.6 ± 11.3 (dw) *	764.9 ± 4.9 (dw)	
Peel		71.4 (fw) *	Nd	
Pulp		67.3 (fw) *	Nd	
Seeds		2819.8 (fw) *	50.4 (fw)	
Cloudberry	*Rubus chamaemorus*	3600 (fw)	-	[36]
		3151 (fw)	-	[35]
Cranberries	*Vaccinium*	120 ± 4 (dw)	-	[54]
Guava	*Psidium guajava* L.	57.2–306 (dw)	-	[55]
Kakadu plum	*Terminalia ferdinandiana*	30,510–140,250 (dw)	-	[37]
		8796.0 ± 156.0 (dw)	6206.0 ± 22.0 (dw)	[38]
Muscadine grapes	*Vitis rotundifolia*	360–912 (fw)	-	[43]
Pomegranate:	*Punica granatum*			[42]
Mesocarp		40,595.4 ± 4434.2 (dw)	234.2 ± 13.0 (dw)	
Peel		43,979.0 ± 394.8 (dw)	637.7 ± 32.8 (dw)	
Red raspberry	*Rubus idaeus*	1500 ± 100 (dw)	-	[54]
		1900–2700 (fw)	-	[36]
		2637–3309 (fw)	-	[35]
Rose hip	*Rosa rugosa*	1096 (fw)	-	[35]
Strawberry	*Fragaria ananassa*	630 ± 90 (dw)	-	[54]
		650–850 (fw)	-	[36]
		683–853 (fw)	-	[35]
**Processed Fruits**				
Pomegranate juice	-	87–2118.3 (mg·L^−1^)	2.1–7.7 (mg·L^−1^)	[42]
Raspberry jam	-	764 (fw)	-	[35]
Strawberry jam	-	245 (fw)	-	[35]
**Seeds and Nuts**				
Pecans	*Carya illinoensis*	330 ± 0.3 (dw)	-	[54]
Walnuts	*Juglans nigra*	590 ± 0.3 (dw)	-	[54]
**Wood**				
Blue gum	*Eucalyptus globulus*	-	500–1700 (dw)	[48]
Common Oak	*Quercus robur*	-	81–228 (dw)	[56]
Pyrenean oak	*Quercus pyrenaica*	-	66–219 (dw)	[56]
Rose gum	*Eucalyptus grandis*	-	280–512 (dw)	[47]
Sessile oak	*Quercus petraea*	-	109–198 (dw)	[56]
Sweet chestnut	*Castanea sativa*	-	74–140 (dw)	[56]
White oak	*Quercus alba*	-	132–277 (dw)	[56]
**Wood bark**				
Blue gum	*Eucalyptus globulus*	-	471 (dw)	[57]
(Hybrid) eucalypt	*Eucalyptus urograndis*	-	2243–2307 (dw)	[58]
Maidens Gum	*Eucalyptus maidenii*	-	1130–1178 (dw)	[58]
Oak	*Quercus robur* + *Quercus petraea*	-	2200–3700 (dw)	[59]
Sweet chestnut	*Castanea sativa*	-	4300–9300 (dw)	[60]
Rose Gum	*Eucalyptus grandis*	-	2639–2721 (dw)	[58]
**Other sources**				
Eucalypt leaves	*Eucalyptus globulus*	3320.0 ± 80.0 (dw)	-	[61]
Filtrates from unbleached kraft wood	*Eucalyptus globulus*	-	98 ± 0.7 (mg/L)	[48]
Sulphite spent liquor	*Eucalyptus globulus*	-	1165.5 (mg/L)	[62]

^#^—total EA after ETs hydrolysis; all values are presented as mg per kg of source (dw = dry weight or fw = full weight). *—Total ET + Total EA derivatives.

The industrial importance of *Eucalyptus* species for cellulosic pulp production in South Europe, Australia, Asia, South America, and South Africa predetermines a particular interest in these angiosperms [63]. Since eucalypt wood is used in pulping processes after the preliminary removal of bark, the latter can be considered as a large source of ETs as well. EA is present in the different industrial streams from the production of both kraft [48] and sulphite [64] pulps. Furthermore, significant amounts of EA and its metal salts, in the form of undesirable waste by-products (pitch deposits, effluents, etc.), are readily available from the pulp industry [18,20,48]. Thus, in addition to fruits, nuts and herbs, the pulping industry can furnish EA in a large scale. Accordingly, contrary to agricultural sources, the pulping industry represents an all-season large-scale underutilized source of EA and its derivatives.

## 4. Production of Ellagic Acid

### 4.1. Extraction from Natural Sources

The recovery and isolation of bioactive phytochemicals from plant materials is usually carried out by employing various extraction procedures. The production of commercial EA is achieved through extraction of the ET-rich plant fraction using acid-methanol mixtures as solvents, followed by ET’s hydrolysis to EA with concentrated HCl or H_2_SO_4_ [65,66]. Most commonly, the composition and the purity of EA/ETs in extracts are monitored by HPLC [32,43,65]. Spectrofluorometric quantification, based on the fluorescence of the EA–borate complex in methanol solutions, can be considered as an alternative and less time-consuming method. It has been successfully used in the determination of EA in brandy [67], pomegranate juice [68] and rosehips [69]. Moreover, a fluorescence nanosensor based on a cyclodextrin–EA–borax complex for food samples was recently described [70]. Advanced mass spectrometry (ESI-MS and MALDI-TOF-MS) and NMR techniques, coupled with liquid chromatography, are used for the confirmation of structural features [71].

Exploring underutilized sources of EA, Lu and Yuan [44] reported a new high-yield process (3.5 g per 100 g pomegranate husk), based on conventional methanol extraction of EA, that utilizes pomegranate husk, a by-product of the pomegranate juice industry. The authors claimed the method was less expensive and suitable for industrial production.

More recently, ionic liquids (ILs) have been applied as alternative solvents for the extraction of value-added compounds from biomass [72]. For instance, Chowdhury and co-workers [73] used a protic and distillable IL [N_1100_][N(C_1_)_2_CO_2_] (*N*,*N*-dimethylammonium *N*′,*N*′-dimethylcarbamate), which improved the solid–liquid extraction (SLE) efficiency of plant materials, resulting in ET fractions containing higher EA concentrations, compared to conventional extraction methods. This SLE method allows the reduction of water content used in the extraction and an easy removal of [N_1100_][N(C_1_)_2_CO_2_] from the system by a low temperature distillation. Another method consisted in using IL microwave-assisted simultaneous extraction and distillation (ILMSHE) of EA, gallic acid and essential oil from the leaves of two *Eucalyptus* species [61]. This ILMSHE process resulted in improved yields, when compared to conventional extraction methods, thus revealing itself as a highly efficient, energy and time saving eco-benign methodology.

It must be noted that, despite being the preferred approaches to EA production, conventional extraction methods are unselective, being affected by the large diversity of ET structures, a vast assortment of plant sources and a troubling purification, thus, leading to low yields of EA, contaminations and excessive costs. Accordingly, there has been a need to develop alternative technologies, more efficient in terms of yield and energy cost, in order to produce EA of higher purity and on a larger scale, ideally from sources that do not compete with the food processing industry. Therefore, biotechnological production has emerged as a promising alternative, being the focus of many studies.

EA can be produced from pomegranate ETs using ellagitannase or ellagitannin acyl hydrolase (EAH) [74]. However, the use of enzymatic methods requires the optimization of production, extraction and enzyme purification. In fact, it is essential to optimize fermentation systems as there are certain limitations: performance ratios of macro and microelements for a given culture media, hidden existence of trace elements and growth factors, and use of unconventional sources of nutrients, among others. Nevertheless, several advances have been made. A possible pathway of ET biodegradation, and the production of EA from punicalagin, was proposed by Ascacio-Valdés and co-authors [75]. Punicalin and gallagic acid would serve as intermediaries. Punicalagin and punicalin from pomegranate husk [76] have been reported as an optimal carbon source for EA production by *Aspergillus niger* GH1, by solid-state fermentation, using a polyurethane foam support [75]. Several studies have attempted to optimize the process for EAH and EA [77,78]. There were also studies that encourage the use of immobilized systems (simulating an immobilization process using fermented solids) in order to improve productivity and efficiency [79,80]. EAH attached to PUF was also produced and applied as a biocatalyst for ET hydrolysis using a continuous system. The authors suggested that the designed device can be used for the development of a large-scale process for continuous ET hydrolysis and EA production [81]. Lastly, a submerged fermentation system, using *Aspergillus fumigatus* and orange peel as the carbon source, was recently described [82].

A kind of semi-synthetic method of EA production from plant materials containing gallates (e.g., tannic acid) was proposed using tannin extraction by different organic solvents, or their mixtures with water, followed by the oxidation of released gallic acid by oxygen or other oxidants under weak alkaline conditions, thus giving rise to EA, which is further purified [83]. Different variations of this approach have been reported recently using different raw materials and purification schemes [84,85].

It was claimed that such processes could be industrially applied. It is worth mentioning that despite the pulping sector being a prospective source for EA derivatives, so far, no reliable solutions were proposed for their recovery from the industrial streams. In fact, there are few published studies that target the isolation of EA from the sulphite liquor from acidic sulphite pulping of *Eucalyptus* wood [62,86]. The main efforts were focused on the extraction of polyphenolic extractives from wood bark to substitute phenol in phenol-formaldehyde resins or to produce fractions of polyphenolics with antioxidant properties [87].

### 4.2. Organic Synthesis

The synthesis of EA and ETs was the topic of several studies [1,88]. Most frequently, the syntheses consisted in the oxidative coupling of galloyls in organic solvent media or, more recently, in ionic liquids. Multistage procedures normally yield a mixture of coupling products that needs a further purification. The most successful syntheses of EA (10–50% yields) via oxidative coupling involve galloyl esters [1,88]. Nevertheless, it must be noted that oxidative aromatic coupling dates back to 1868, with the Löwe synthesis of ellagic acid by heating gallic acid with arsenic acid or silver oxide [89].

The synthesis of ETs is much more complex than that of EA, has relatively small yields and poses a major challenge: generating stereoselective linkages of galloyl-derived biphenyl moieties across different sites of the glucose core [1]. Nevertheless, several total syntheses have been reported over the years [90,91,92,93,94].

Overall, due to the peculiar chemical and structural features of EA/ETs in terms of chemoselectivity, regioselectivity, and stereoselectivity, organic synthesis′ selectivity challenges of EA/ETs, coupled with a rising preference for natural food additives and lingering consumer suspicion of synthetic substitutes, encourage the production and utilization of all-natural antioxidants.

## 5. Technical Applications of Ellagic Acid

The major applications of EA and its derivatives are limited to medicinal and nutritional purposes. Nevertheless, in recent years, more studies have been contemplating different technical applications. Thus, due to its particular chemical and structural features, EA reveals prospective industrial significance for the synthesis of new bioengineered materials. Zhang and co-workers [16] reported the synthesis of a macroporous ellagitannic acid ion-exchange resin for the easy removal of Cu^2+^, Fe^3+^, Ce^3+^ and La^3+^ from solutions. Later, Przewloka and Shearer [15] reported EA and water-soluble ellagates’ utilization for the removal of divalent ionic metal ions from aqueous solution, confirming the potential of EA and its derivatives as metal chelants. Furthermore, Reitze, in collaboration with Przewloka and Shearer [14], reported the synthesis of several potential EA-based polymer precursors, including monomers and oligomers, offering new options for polymer applications.

More recently, Wang and co-workers [95] developed conductivity-based sensors via the assembling of EA molecules through π-π interaction and hydrogen bonding between EA molecules. Due to the near planar structure of EA, the obtained nanostructures exhibit a 1D dimensional structure, whose conductivity and fluorescence selectively change in the presence of nitrobenzene, indicating the potential of these nanomaterials for the detection of explosive chemicals. EA and catechols, in combination with lignin, were reported as a part of all-solid potentiometric chemical sensor for the selective detection of Cu^2+^ in aqueous solutions [96]. The sensing membrane, composed of tannin-lignin-based polyurethane doped by multi-walled carbon nanotubes (MWCNT), demonstrated long-term stability. It has been suggested that EA and catechol play a determining role in the specific chelation of Cu^2+^, contributing to the ionic sensing mechanism. According to the results of another work, due to good redox properties and high thermal stability (up to 400 °C), EA (50 wt.%) mixed with acetylene black (40 wt.%) and polyvinylidene fluoride (PVDF, 10 wt.%), resulted in an efficient organic electrode material for rechargeable Li-ion batteries [17].

Barnaby and co-workers [97] reported the biomimetic synthesis of shape-controlled Ag-nanoparticles (NPs) in the presence of EA as the chelating agent. These EA-based Ag-NPs complexes exhibited enhanced antibacterial properties when compared to Ag-NPs or EA used separately. The development of the Ag nanochains in the presence of EA was achieved by a template free method without the need for high temperatures or reducing agents. Furthermore, the production of EA-based microassemblies, used afterwards as templates for the growth of CdSe nanoparticles, was reported [98]. The thermal stability and efficiency of these EA-based nanocomposites to photodegrade alizarin red (a model toxic aromatic compound) was confirmed, suggesting that these nanocomposites have potential applications in the degradation of environmental pollutants such as toxic aromatic compounds.

In order to enhance EA bioavailability and maximize its activity, attempts have been made to develop a delivery system using a chitosan polymer in composite films [99], collagen and chitosan-based scaffolds [100] and nanocapsules [101,102]. Apart from biomedical applications, more recently, Vilela and co-workers [103] have proposed chitosan/EA films as promising eco-friendly active food packaging material. Another interesting application of EA is for copigmentation in enhancement of color properties in wines [104]. Apparently, the technical applications of EA and its derivatives can be expanded as their availability in the market increases.

## 6. Bioavailability of Ellagitannins and Ellagic Acid

**Figure 3:**
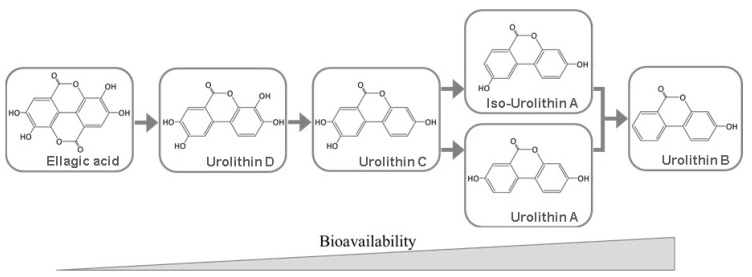
Urolithins derived from ellagic acid and their relative bioavailability.

There are numerous factors that can influence EA bioavailability: low solubility in aqueous media under gastric conditions, *in vivo* hydrolysis of ETs to release EA, the type of ET as EA precursor, limited intestinal absorption and/or transport and the catabolism of EA by the gut microbiota to produce urolithins. Furthermore, EA pharmacokinetics revealed high inter-individual variability [105]. In addition, González-Sarrías and coworkers [105] conclude that EA’s bioavailability is not enhanced by a higher intake and hardly exceeds 100 nM in human plasma. Conversely, urolithins can attain bloodstream concentrations at the micromolar level [106]. As previously mentioned, ETs are hydrolyzed to EA and the latter is either absorbed or transformed via lactone-ring cleavage, decarboxylation, and, after that, de-hydroxylation reactions resulting in dibenzo[*b,d*]pyran-6-one derivatives, with different phenolic hydroxylation patterns, known as urolithins (Figure 3). Methylated and glucuronidated counterparts such as urolithin A glucuronide, urolithin-C glucuronide, urolithin-C methyl ether glucuronide, and dimethyl ellagic acid glucuronide have been found in human plasma after the consumption of different sources of ellagitannins [107]. Urolithins have a higher bioavailability and it is debatable whether urolithins formed *in vivo* are the main reason for the effects attributed to the ETs [106]. Given this background, it should be considered that cultured cells representing systemic tissues and organs may not be in direct contact *in vivo* with food ETs or EA [108]. In fact, it can be the cause of discrepancies between *in vitro* and *in vivo* results, which can also be linked to the inter-individual variability in quality and quantity of urolithin production [109].

In order to counter these flaws, various studies have been made to enhance EA solubility, and several strategies for EA delivery systems were developed over the years [27,99,101,102,110,111,112,113,114,115]. For example, by complexing EA with cyclodextrins, it is possible to enhance EA’s anti-inflammatory, antioxidant or even antimicrobial properties [111,114]. Additionally, the production of several urolithins, such as M5, M6, C, [116] isourolithin A [117] and urolithins A and B [118], has been described. The possibilities of such probiotics mitigate EA’s bioavailability limitations and bolster applications of EA in the development of functional foods and nutraceuticals. The formation of urolithin-like compounds is also possible under conditions of alkaline pulping at relatively high pH and temperature [19].

## 7. Biological Functionality of Ellagic Acid and Its Derivatives

### 7.1. Antioxidant Properties

**Figure 4:**
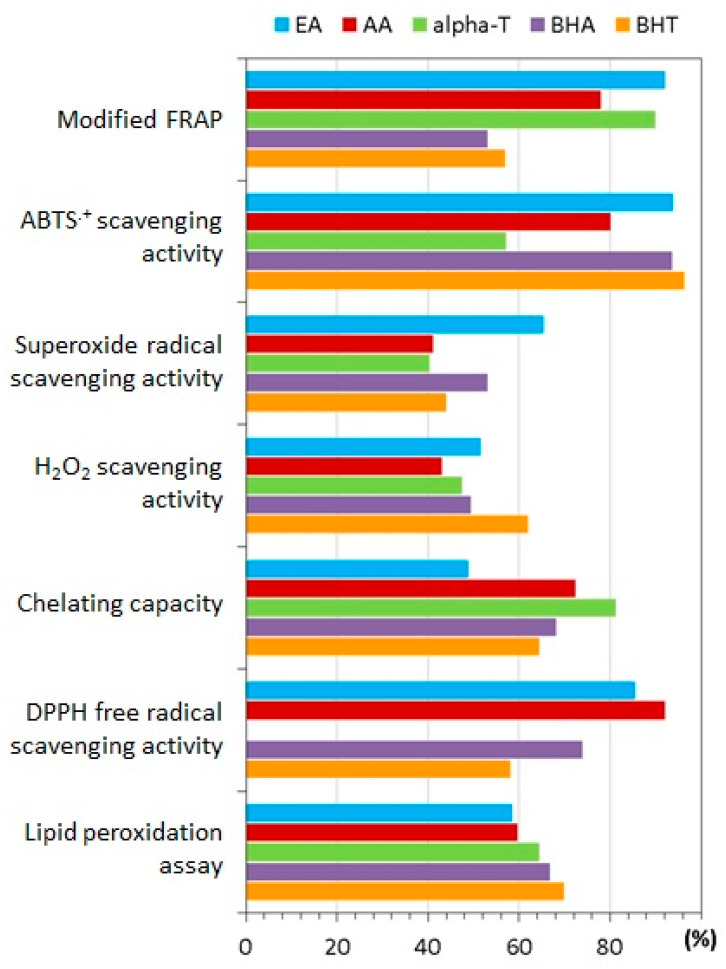
*In vitro* radical scavenging and antioxidant capacity studies of ellagic acid (EA) vs. butylated hydroxyanisole (BHA), butylated hydroxytoluene (BHT), alfa-tocopherol (alpha-T) and ascorbic acid (AA). The values are presented in percentages at the same concentrations, except for the modified FRAP test, which indicates the absorbance of the reaction mixture (adapted from [120]).

Through the inhibitory effects of several tannins on the cupric ion-catalyzed autoxidation of ascorbic acid, it was demonstrated that the antioxidant activity of ETs and EA is higher than that of polyphenols of low molecular weight [32]. It is important to mention that in spite of its rather small size, EA exhibited high antioxidant capacity due to the stability of its free radical [119]. Kilic and co-workers [120] performed several *in vitro* assays comparing EA to standard antioxidants such as butylated hydroxyanisole, butylated hydroxytoluene, alfa-tocopherol and ascorbic acid (Figure 4). Among the results, EA was found to be a potential antioxidant for the prevention of lipid oxidation in food products.

Free radical-mediated oxidative damage of biological molecules has been associated with pathogenesis of several diseases and senescence physiological pathways [121,122,123,124]. Given their ability to quench free electrons, EA and ET can protect vital molecules from hazardous free radicals [125]. Additionally, due to structural similarities with several key signaling molecules, they can trigger gene expression of molecules engaged in antioxidant defense. Similarly, they can repress the expression of proteins, enzymes and transcription factors involved in oxidative stress-producing pathways, such as nicotinamide adenine dinucleotide phosphate-oxidase (NADPH) and Cytochrome P450 (CYP)-dependent phase-I enzymes. In such a way, EA participates not only in the management of endogenous and exogenous sources of reactive oxygen species (ROS) but also in antioxidant defense mechanisms [126]. A more detailed overview of EA’s antioxidant mechanisms and their link with multiple biological activities can be found in a couple of recent reviews [127,128].

### 7.2. Biological Activity

The above-mentioned functions have a vital role in maintaining cellular homeostasis and bestowing these compounds with preventive and protective properties in many biological systems and cell types. Table 2 presents, in summary, some potential biomedical applications of EA, ETs and derivatives. From the studies of the past decade, it is clear that there is a diversity of possible applications of EA and ETs in biomedical/pharmaceutical fields. An in-depth review of the pharmacological properties of ellagic acid can be found in a 2018 paper of Rios et al. [129].

There is still a focus on EA as a test compound and the results are promising, however EA derivatives and ETs have also been thoroughly investigated and it seems that they are yet to reveal their full potential. Reports of their activity on pathogens include infectious agents such as bacterium, virus, fungus and even protozoa [110,114,130,131,132,133,134,135,136]. Remarkably, prebiotic effects were also registered [130]. It is worth noting that C-glucosidic ellagitannins, active against Acyclovir (ACV)—resistant strains of the *Herpes simplex* virus, exhibited synergistic effects when used in combination with ACV [136]. It should also be noted that pre-treatment with ellagitannin-rich fraction obtained from *Eucalyptus citriodora*, at a dose of 100 mg/kg, resulted in higher gastroprotection (99.6% in ethanol-induced acute gastric ulceration) than that of the omeprazole, a widely known proton pump inhibitor. Notably, the authors point out that ETs were found to be the major active components responsible for the marked antioxidant, anti-inflammatory and gastroprotective properties [139].

Additionally, the involvement of EA/ETs in the GABAergic system, inhibition of key enzymes such as aldose reductase, acethylcholinesterase and protein tyrosine phosphatases, suppression of pro-inflammatory markers, and interaction with adrenergic and serotonergic systems, establish a solid foundation for possible breakthroughs in treatments and/or prevention of many illnesses and related clinical complications [9,10,12,13,138]. As an example, some recent studies suggest EA can act as an inhibitor of acetylcholinesterase, thus increasing acetylcholine brain levels. As a result, there is a possibility to partly minimize or restore the impairment of cognitive dysfunctions in neurodegenerative pathologies such as Alzheimer’s disease [12,13]. Finally, one of the most paradigmatic activities of EA, melanogenesis inhibition, was reported to occur partially due to EA’s antioxidant power [142].

In accordance with the foregoing, EA and its derivatives can be considered for eventual exploitation in supplement and functional food industries, mostly due to their preventive activities in various cell systems. The development of pharmaceuticals prompts further research, since it will be mainly conditioned by the enhancement of EA bioavailability via delivery systems.

## 8. Concluding Remarks

EA and its derivatives are natural bioactive compounds with significant beneficial health effects and potential for advanced technical applications. However, the expansion of application areas and the commercial exploration of EA are hampered by the lack of market availability of high-grade products. Accordingly, the wood processing sector, in particular by-products from the pulping industry, could be a valuable all-season source alternative to food competing crops. The detailed knowledge on structural changes of ETs and the composition of EA derivatives in industrial streams would be the first step towards a wider exploration in technical and biomedical applications.

## Figures and Tables

**Table 2:** Possible biological effects of EA and its derivatives.

Activity	Active Compound	Main Features	Ref.
Antibacterial (Gram-Positive)	Commercial extract of pomegranate byproduct (POMx) and punicalagin	Inhibited the growth of pathogenic *Clostridium* and *Staphyloccocus aureus*	[130]
Antibacterial (Gram-Positive)	Ellagic acid	Action against *Bacillus luteus* and *Listeria monocytogenes*	[114]
Antibacterial (Gram-Negative)	Tellimagrandin I	Time- and dose-dependent bactericidal activity against *Helicobacter pylori*	[131]
Antibacterial (Gram-Negative)	Ellagic acid	EA—cyclodextrin complex expressed activity against *Escherichia coli* and *Pseudomonas aeruginosa*	[114]
Antimycobacterial	Punicalagin	Inhibited the growth of *Mycobacterium tuberculosis* typus humanus ATCC 27294 and patient strain of *Mycobacterium tuberculosis* sensitive to the standard antituberculosis drugs	[132]
Antileishmanial	Geraniin, phyllanthusiin B and elaeocarpusin	Exhibited effect against protozoa *Leishmania donovani,* comparable to that of the amphotericin B	[133]
Antimalarial	Ellagic acid	*In vitro* against all *Plasmodium falciparum* strains. *In vivo* against *Plasmodium vinckei petteri*; potentiates the activity of chloroquine, mefloquine, artesunate and atovaquone	[134]
Antibabesial	Ellagic acid	*In vivo* against *Babesia microti;* EA nanoparticles as an alternative antiparasitic agent	[110]
Antifungal	Candelitannin (ellagitannin) isolated from *E. antisyphilitica* Zucc.	Effective against *Alternaria alternata*, *Fusarium oxyzporum*, *Colletotrichum gloeosporoides* and *Rhizoctnia solani*	[135]
Antifungal	Ellagic acid	Action against *Candida albicans*	[114]
Antiviral	Castalagin, vescalagin and grandinin.	Action against acyclovir (ACV)—resistant strains of *Herpes simplex* virus HSV^−1^ and HSV-2; synergistic effects when used in combination with ACV	[136]
Prebiotic effect	Commercial extract of pomegranate byproduct (POMx) and punicalagin	Enhanced growth of *Bifidobacterium breve* and *Bifidobacterium infantis*	[130]
Anti-inflammatory	Ellagic acid, gallic acid and punicalagin A&B	Potential inhibition of LPS-induced NO, PGE-2 and IL-6 production	[137]
Anti-inflammatory	Ellagic acid	Enhancement of EA’s anti-inflammatory properties *in vivo* by inclusion complex of EA with hydroxypropyl-β-cyclodextrin	[111]
Treatment of Type 2 diabetes mellitus	Ellagic acid and ETs from *Agrimonia pilosa* Ledeb.	Inhibition of protein tyrosine phosphatases (PTP1B)	[13]
Prevention of diabetic complications	Ellagic acid	ALR2 (aldose reductase) inhibition and antiglycating effect of EA could possibly delay progression of cataract	[138]
Anticancerous agent	Ellagic acid	Inhibition of SphK1 (sphingosine kinase 1)	[11]
Antiangiogenic and antiproliferative effect	Ellagic acid	Reduction in metastatic potential of bladder cancer and enhancement of the efficacy of anti-VEGF-A therapies	[7]
Gastroprotective	Ellagitannin-rich fraction obtained from *E. citriodora*	Possibly due to their antioxidant, anti-inflammatory and anti-apoptotic properties. Partially mediated by attenuating induced oxidative stress and by the reduction of pro-inflammatory markers.	[139]
Hepatoprotective	Ellagic acid	Suppression of caspase-3, bcl-2, NF-kB and Nrf-2	[6]
Antiarrhythmic	Ellagic acid	Antilipid peroxidation property and antihyperlipidemic activity through 3-hydroxy-3 methyl glutaryl CoA reductase inhibition; cardioprotective effect	[140]
Antiasthmatic	*L. pacari* extract and ellagic acid	Effective eosinophilic inflammation suppressors	[141]
Antihyperlipidemic	Ellagic acid	EA-CoQ10 nanoparticles effectively attenuated induced hyperlipidemia in rats	[112]
Antiepileptic	Ellagic acid	Possibly achieved through increase of brain GABA levels	[9]
Antianxiety	Ellagic acid	Possible involvement of GABAergic system in the anxiolytic action	[10]
Antidepressant	Ellagic acid	Possible interaction through adrenergic and serotonergic systems or through inhibition of inducible NOS	[8]
Neuroprotective in SAD	Ellagic acid	Diminished oxidative stress profile, pro-inflammatory markers, acetylcholinesterase activity, and amyloid-β plaque level in induced SAD (Sporadic Alzheimer’s Disease) rats	[12]
Skin-whitening agent	Ellagic acid	EA acts as an alternative substrate of tyrosinase, inhibiting the melanogenesis process	[142]

## References

[1] Quideau S., Feldman K.S. (1996). Ellagitannin chemistry. Chem. Rev..

[2] Khanbabaee K., van Ree T. (2001). Tannins: Classification and definition. Nat. Prod. Rep..

[3] Yamada H., Wakamori S., Hirokane T., Ikeuchi K., Matsumoto S. (2018). Structural revisions in natural ellagitannins. Molecules.

[4] Covington A.D. (1997). Modern tannins chemistry. Chem. Soc. Rev..

[5] Wu X., Gu L., Holden J., Haytowitz D.B., Gebhardt S.E., Beecher G., Prior R.L. (2004). Development of a database for total antioxidant capacity in foods: A preliminary study. J. Food Compos. Anal..

[6] Aslan A., Gok O., Erman O., Kuloglu T. (2018). Ellagic acid impedes carbontetrachloride-induced liver damage in rats through suppression of NF-kB, Bcl-2 and regulating Nrf-2 and caspase pathway. Biomed. Pharmacother..

[7] Ceci C., Tentori L., Atzori M.G., Lacal P.M., Bonanno E., Scimeca M., Cicconi R., Mattei M., de Martino M.G., Vespasiani G. (2016). Ellagic acid inhibits bladder cancer invasiveness and *in vivo* tumor growth. Nutrients.

[8] Dhingra D., Chhillar R. (2012). Antidepressant-like activity of ellagic acid in unstressed and acute immobilization-induced stressed mice. Pharmacol. Rep..

[9] Dhingra D., Jangra A. (2014). Antiepileptic activity of ellagic acid, a naturally occurring polyphenolic compound, in mice. J. Funct. Foods.

[10] Girish C., Raj V., Arya J., Balakrishnan S. (2013). Involvement of the GABAergic system in the anxiolytic-like effect of the flavonoid ellagic acid in mice. Eur. J. Pharmacol..

[11] Gupta P., Mohammad T., Khan P., Alajmi M.F., Hussain A., Rehman M.T., Hassan M.I. (2019). Evaluation of ellagic acid as an inhibitor of sphingosine kinase 1: A targeted approach towards anticancer therapy. Biomed. Pharmacother..

[12] Jha A.B., Panchal S.S., Shah A. (2018). Ellagic acid: Insights into its neuroprotective and cognitive enhancement effects in sporadic Alzheimer’s disease. Pharmacol. Biochem. Behav..

[13] Nguyen D.H., Seo U.M., Zhao B.T., Le D.D., Seong S.H., Choi J.S., Min B.S., Woo M.H. (2017). Ellagitannin and flavonoid constituents from *Agrimonia pilosa* Ledeb. with their protein tyrosine phosphatase and acetylcholinesterase inhibitory activities. Bioorg. Chem..

[14] Reitze J.D., Przewloka S.R., Shearer B.J. (2001). The further chemistry of ellagic acid I. Synthesis of tetramethylellagic acid and associated polymer precursors. Holzforschung.

[15] Przewloka S.R., Shearer B.J. (2002). The further chemistry of ellagic acid II. Ellagic acid and water-soluble ellagates as metal precipitants. Holzforschung.

[16] Zhang N.Z., Chen Y.Y. (1988). Synthesis of macroporous ellagitannic acid resin and its chelating properties for metal ions. J. Macromol. Sci. Part A Chem..

[17] Goriparti S., Harish M.N.K., Sampath S. (2013). Ellagic acid—a novel organic electrode material for high capacity lithium ion batteries. Chem. Commun..

[18] Gardner J.A.F., Hillis W.E., Hillis W.E. (1962). The influence of extractives on the pulping of wood. Wood Extractives and Their Significance to the Pulp and Paper Industries.

[19] Hemingway R.W., Hillis W.E. (1971). Behavior of ellagitannins, gallic acid, and ellagic acid under alkaline conditions. TAPPI J..

[20] Sjöström J., Bädenlid R., Norborg M.A. (1993). Short note: Analysis of ellagic acid in pulp mill deposits. Holzforschung.

[21] Pinto P.C.R., Sousa G., Crispim F., Silvestre A.J.D., Neto C.P. (2013). *Eucalyptus globulus* bark as source of tannin extracts for application in leather industry. ACS Sustain. Chem. Eng..

[22] Braconnot H. (1818). Observations sur la préparation et la purification de l′acide gallique, et sur l′existence d′un acide nouveau dans la noix de galle. Ann. Chim. Phys..

[23] Berzelius J.J., Esslinger M., Hoefer F. (1849). Acide ellagique (*Acidum ellagicum*) (I). Traité de Chimie Minerale, Végétale et Animale.

[24] Mathieson A.M., Poppleton B.J. (1968). The crystal structure of ellagic acid. Acta Crystallogr. Sect. B Struct. Crystallogr. Cryst. Chem..

[25] Rossi M., Erlebacher J., Zacharias D.E., Carrell H.L., Iannucci B. (1991). The crystal and molecular structure of ellagic acid dihydrate: A dietary anti-cancer agent. Carcinogenesis.

[26] Li X.C., Elsohly H.N., Hufford C.D., Clark A.M. (1999). NMR assignments of ellagic acid derivatives. Magn. Reson. Chem..

[27] Bala I., Bhardwaj V., Hariharan S., Kumar M.N.V.R. (2006). Analytical methods for assay of ellagic acid and its solubility studies. J. Pharm. Biomed. Anal..

[28] Musialik M., Kuzmicz R., Pawcowski T.S., Litwinienko G. (2009). Acidity of hydroxyl groups: An overlooked influence on antiradical properties of flavonoids. J. Org. Chem..

[29] Simić A.Z., Verbić T.Ž., Sentić M.N., Vojić M.P., Juranić I.O., Manojlović D.D. (2013). Study of ellagic acid electro-oxidation mechanism. Monatsh. Chem..

[30] Marković Z., Milenković D., Đorović J., Dimitrić Marković J.M., Lučić B., Amić D. (2013). A DFT and PM6 study of free radical scavenging activity of ellagic acid. Monatsh. Chem. Chem. Mon..

[31] Nenadis N., Tsimidou M.Z. (2012). Contribution of DFT computed molecular descriptors in the study of radical scavenging activity trend of natural hydroxybenzaldehydes and corresponding acids. Food Res. Int..

[32] Okuda T., Yoshida T., Hatano T., Ito H., Quideau S. (2009). Ellagitannins renewed the concept of tannins. Chemistry and Biology of Ellagitannins: An Underestimated Class of Bioactive Plant Polyphenols.

[33] Quideau S., Jourdes M., Saucier C., Glories Y., Pardon P., Baudry C. (2003). DNA topoisomerase inhibitor acutissimin a and other flavano-ellagitannins in red wine. Angew. Chem. Int. Ed. Engl..

[34] Niemetz R., Gross G.G. (2005). Enzymology of gallotannin and ellagitannin biosynthesis. Phytochemistry.

[35] Koponen J.M., Happonen A.M., Mattila P.H., Törrönen A.R. (2007). Contents of anthocyanins and ellagitannins in selected foods consumed in Finland. J. Agric. Food Chem..

[36] Määttä-Riihinen K.R., Kamal-Eldin A., Törrönen A.R. (2004). Identification and quantification of phenolic compounds in berries of *Fragaria* and *Rubus* species (family Rosaceae). J. Agric. Food Chem..

[37] Konczak I., Maillot F., Dalar A. (2014). Phytochemical divergence in 45 accessions of *Terminalia ferdinandiana* (Kakadu plum). Food Chem..

[38] Williams D.J., Edwards D., Pun S., Chaliha M., Sultanbawa Y. (2014). Profiling ellagic acid content: The importance of form and ascorbic acid levels. Food Res. Int..

[39] Fukuda T., Ito H., Yoshida T. (2003). Antioxidative polyphenols from walnuts (*Juglans regia* L.). Phytochemistry.

[40] Villarreal-Lozoya J.E., Lombardini L., Cisneros-Zevallos L. (2007). Phytochemical constituents and antioxidant capacity of different pecan [*Carya illinoinensis* (Wangenh.) K. Koch] cultivars. Food Chem..

[41] Fracassetti D., Costa C., Moulay L., Tomás-Barberán F.A. (2013). Ellagic acid derivatives, ellagitannins, proanthocyanidins and other phenolics, vitamin C and antioxidant capacity of two powder products from camu-camu fruit (*Myrciaria dubia*). Food Chem..

[42] Fischer U.A., Carle R., Kammerer D.R. (2011). Identification and quantification of phenolic compounds from pomegranate (*Punica granatum* L.) peel, mesocarp, aril and differently produced juices by HPLC-DAD-ESI/MS^n^. Food Chem..

[43] Lee J.-H., Johnson J.V., Talcott S.T. (2005). Identification of ellagic acid conjugates and other polyphenolics in muscadine grapes by HPLC-ESI-MS. J. Agric. Food Chem..

[44] Lu J., Yuan Q. (2008). A new method for ellagic acid production from pomegranate husk. J. Food Process. Eng..

[45] Hillis W.E., Hillis W.E. (1962). The distribution and formation of polyphenols within the tree. Wood Extractives and Their Significance to the Pulp and Paper Industries.

[46] Santos S.A.O., Villaverde J.J., Sousa A.F., Coelho J.F.J., Neto C.P., Silvestre A.J.D. (2013). Phenolic composition and antioxidant activity of industrial cork by-products. Ind. Crops Prod..

[47] Santos S.A.O., Vilela C., Domingues R.M.A., Oliveira C.S.D., Villaverde J.J., Freire C.S.R., Neto C.P., Silvestre A.J.D. (2017). Secondary metabolites from *Eucalyptus grandis* wood cultivated in Portugal, Brazil and South Africa. Ind. Crops Prod..

[48] Costa E.V., Lima D.L.D., Evtyugin D.V., Esteves V.I. (2014). Development and application of a capillary electrophoresis method for the determination of ellagic acid in *E. globulus* wood and in filtrates from *E. globulus* kraft pulp. Wood Sci. Technol..

[49] Conde E., Cadahia E., Garciavallejo M., Tomasbarberan F. (1995). Low molecular weight polyphenols in wood and bark of *Eucalyptus globulus*. Wood Fiber Sci..

[50] Charrier B., Marques M., Haluk J.P. (1992). HPLC analysis of gallic and ellagic acids in european oakwood (*Quercus robur* L.) and eucalyptus (*Eucalyptus globulus*). Holzforschung.

[51] Elgailani I.E.H., Ishak C.Y. (2014). Determination of tannins of three common *Acacia* species of Sudan. Adv. Chem..

[52] Sanz M., Cadahía E., Esteruelas E., Muñoz Á.M., Fernández De Simón B., Hernández T., Estrella I. (2010). Phenolic compounds in chestnut (*Castanea sativa* Mill.) heartwood. Effect of toasting at cooperage. J. Agric. Food Chem..

[53] Fengel D., Wegener G. (1989). Extractives. Wood—Chemistry, Ultrastructure, Reactions.

[54] Daniel E.M., Krupnick A.S., Heur Y.H., Blinzler J.A., Nims R.W., Stoner G.D. (1989). Extraction, stability, and quantitation of ellagic acid in various fruits and nuts. J. Food Compos. Anal..

[55] dos Santos W.N.L., da Silva Sauthier M.C., dos Santos A.M.P., de Andrade Santana D., Azevedo R.S.A., da Cruz Caldas J. (2017). Simultaneous determination of 13 phenolic bioactive compounds in guava (*Psidium guajava* L.) by HPLC-PAD with evaluation using PCA and Neural Network Analysis (NNA). Microchem. J..

[56] Alañón M.E., Castro-Vázquez L., Díaz-Maroto M.C., Hermosín-Gutiérrez I., Gordon M.H., Pérez-Coello M.S. (2011). Antioxidant capacity and phenolic composition of different woods used in cooperage. Food Chem..

[57] Santos S.A.O., Freire C.S.R., Domingues M.R.M., Silvestre A.J.D., Neto C.P. (2011). Characterization of phenolic components in polar extracts of *Eucalyptus globulus* Labill. bark by high-performance liquid chromatography-mass spectrometry. J. Agric. Food Chem..

[58] Santos S.A.O., José J., Freire C.S.R., Domingues M.R.M., Pascoal C., Silvestre A.J.D. (2012). Phenolic composition and antioxidant activity of *Eucalyptus grandis*, *E. urograndis* (*E. grandis × E. urophylla*) and *E. maidenii* bark extracts. Ind. Crop. Prod..

[59] Dedrie M., Jacquet N., Bombeck P.L., Hébert J., Richel A. (2015). Oak barks as raw materials for the extraction of polyphenols for the chemical and pharmaceutical sectors: A regional case study. Ind. Crops Prod..

[60] Comandini P., Lerma-García M.J., Simó-Alfonso E.F., Toschi T.G. (2014). Tannin analysis of chestnut bark samples (*Castanea sativa* Mill.) by HPLC-DAD-MS. Food Chem..

[61] Liu Z., Chen Z., Han F., Kang X., Gu H., Yang L. (2016). Microwave-assisted method for simultaneous hydrolysis and extraction in obtaining ellagic acid, gallic acid and essential oil from *Eucalyptus globulus* leaves using Brönsted acidic ionic liquid [HO_3_S(CH_2_)_4_mim]HSO_4_. Ind. Crops Prod..

[62] Alexandri M., Papapostolou H., Vlysidis A., Gardeli C., Komaitis M., Papanikolaou S., Koutinas A.A. (2016). Extraction of phenolic compounds and succinic acid production from spent sulphite liquor. J. Chem. Technol. Biotechnol..

[63] Rana V., Joshi G., Singh S.P., Gupta P.K., Bhojvaid P.P., Kaushik S., Singh Y.P., Kumar D., Thapliyal M., Barthwal S. (2014). Eucalypts in pulp and paper industry. Eucalypts in India.

[64] Rodrigues P.F., Evtyugin D.D., Evtuguin D.V., Prates A. (2018). Extractives profiles in the production of sulphite dissolving pulp from *Eucalyptus globulus* wood. J. Wood Chem. Technol..

[65] Lei Z., Jervis J., Helm R.F. (2001). Use of methanolysis for the determination of total ellagic and gallic acid contents of wood and food products. J. Agric. Food Chem..

[66] Wilson T.C., Hagerman A.E. (1990). Quantitative determination of ellagic acid. J. Agric. Food Chem..

[67] Sádecká J., Tóthová J. (2012). Spectrofluorimetric determination of ellagic acid in brandy. Food Chem..

[68] Huerga-González V., Lage-Yusty M.A., Lago-Crespo M., López-Hernández J. (2015). Comparison of methods for the study of ellagic acid in pomegranate juice beverages. Food Anal. Methods.

[69] Szmagara A., Krzyszczak A., Sadok I., Karczmarz K., Staniszewska M.M., Stefaniak E.A. (2019). Determination of ellagic acid in rose matrix by spectrofluorimetry. J. Food Comp. Anal..

[70] Matencio A., Navarro-Orcajada S., García-Carmona F., López-Nicolás J.M. (2018). Ellagic acid—borax fluorescence interaction: Application for novel cyclodextrin-borax nanosensors for analyzing ellagic acid in food samples. Food Funct..

[71] Kool M.M., Comeskey D.J., Cooney J.M., McGhie T.K. (2010). Structural identification of the main ellagitannins of a boysenberry (*Rubus loganbaccus × baileyanus* Britt.) extract by LC-ESI-MS/MS, MALDI-TOF-MS and NMR spectroscopy. Food Chem..

[72] Passos H., Freire M.G., Coutinho J.A.P. (2014). Ionic liquid solutions as extractive solvents for value-added compounds from biomass. Green Chem..

[73] Chowdhury S.A., Vijayaraghavan R., MacFarlane D.R. (2010). Distillable ionic liquid extraction of tannins from plant materials. Green Chem..

[74] Robledo A., Aguilera-Carbó A., Rodriguez R., Martinez J.L., Garza Y., Aguilar C.N. (2008). Ellagic acid production by *Aspergillus niger* in solid state fermentation of pomegranate residues. J. Ind. Microbiol. Biotechnol..

[75] Ascacio-Valdés J.A., Buenrostro J.J., De la Cruz R., Sepúlveda L., Aguilera A.F., Prado A., Contreras J.C., Rodríguez R., Aguilar C.N. (2014). Fungal biodegradation of pomegranate ellagitannins. J. Basic Microbiol..

[76] Seeram N., Lee R., Hardy M., Heber D. (2005). Rapid large scale purification of ellagitannins from pomegranate husk, a by-product of the commercial juice industry. Sep. Purif. Technol..

[77] Aguilera-Carbo A., Hernández J.S., Augur C., Prado-Barragan L.A., Favela-Torres E., Aguilar C.N. (2009). Ellagic acid production from biodegradation of creosote bush ellagitannins by *Aspergillus niger* in solid state culture. Food Bioprocess. Technol..

[78] Sepúlveda L., Aguilera-Carbó A., Ascacio-Valdés J.A., Rodríguez-Herrera R., Martínez-Hernández J.L., Aguilar C.N. (2012). Optimization of ellagic acid accumulation by *Aspergillus niger* GH1 in solid state culture using pomegranate shell powder as a support. Process. Biochem..

[79] Hu T., Zhou Y., Dai L., Wang Y., Liu D., Zhang J., Liu H. (2011). Enhanced cellulase production by solid state fermentation with polyurethane foam as inert supports. Procedia Eng..

[80] Silva M.F., Rigo D., Mossi V., Dallago R.M., Henrick P., Kuhn G.D.O., Rosa C.D., Oliveira D., Oliveira J.V., Treichel H. (2013). Evaluation of enzymatic activity of commercial inulinase from *Aspergillus niger* immobilized in polyurethane foam. Food Bioprod. Process..

[81] Buenrostro-Figueroa J., Huerta-Ochoa S., Prado-Barragán A., Ascacio-Valdés J., Sepúlveda L., Rodríguez R., Aguilera-Carbó A., Aguilar C.N. (2014). Continuous production of ellagic acid in a packed-bed reactor. Process. Biochem..

[82] Sepúlveda L., Laredo-Alcalá E., Buenrostro-Figueroa J.J., Ascacio-Valdés J.A., Genisheva Z., Aguilar C., Teixeira J. (2020). Ellagic acid production using polyphenols from orange peel waste by submerged fermentation. Electron. J. Biotechn..

[83] Mizusawa K., Imai Y., Yuasa K., Koyama H., Yamaji N., Kataoka S., Oguma T. (1993). Process for Producing Ellagic Acid. US Patent.

[84] Hongwei L., Minhua Y., Yanming Y., Fan L., Hai G., Qingqing Z. (2018). Method for Preparing Ellagic Acid from Tara Seeds Pod. CN Patent.

[85] Yefu M. (2015). Method and Device for Preparation of Ellagic Acid from Gall Flowers. CN Patent.

[86] Llano T., Alexandri M., Koutinas A.A., Gardeli C., Papapostolou H., Coz A., Quijorna N., Andres A., Komaitis M. (2015). Liquid–liquid extraction of phenolic compounds from spent sulphite liquor. Waste Biomass Valor..

[87] Pizzi A., Belgacem M.N., Gandini A. (2008). Tannins: Major sources, properties and applications. Monomers, Polymers and Composites from Renewable Resources.

[88] Chowdhury S.A., Dean P.M., Vijayaraghavan R., MacFarlane D.R. (2011). Efficient synthesis of ellagic acid salts using distillable ionic liquids. Aust. J. Chem..

[89] Löwe J. (1868). Ueber die bildung von ellagsäure aus gallussäure. J. prakt. Chem..

[90] Yamada H., Nagao K., Dokei K., Kasai Y., Michihata N. (2008). Total synthesis of (–)-Corilagin. J. Am. Chem. Soc..

[91] Yamada H., Ohara K., Ogura T. (2013). Total synthesis of Cercidinin A. Eur. J. Org. Chem..

[92] Yamaguchi S., Ashikaga Y., Nishii K., Yamada H. (2012). Total synthesis of the proposed structure of roxbin B; the nonidentical outcome. Org. Lett..

[93] Yamaguchi S., Hirokane T., Yoshida T., Tanaka T., Hatano T., Ito H., Nonaka G.-I., Yamada H. (2013). Roxbin B is cuspinin: Structural revision and total synthesis. J. Org. Chem..

[94] Richieu A., Peixoto P., Pouységu L., Denis D., Quideau S. (2017). Bio-inspired total synthesis of (−)-Vescalin, a nonahydroxytriphenoylated *C*-glucosidic ellagitannin. Angew. Chem. Int. Ed..

[95] Wang H., Xu X., Lee C., Johnson C., Sohlberg K., Ji H.F. (2012). Highly selective sensing of nitroaromatics using nanomaterials of ellagic acid. J. Phys. Chem. C.

[96] Gonçalves S.S.L., Rudnitskaya A., Sales A.J.M., Costa L.M.C., Evtuguin D.V. (2020). Nanocomposite Polymeric Materials Based on Eucalyptus Lignoboost^®^ Kraft Lignin for Liquid Sensing Applications. Materials.

[97] Barnaby S.N., Yu S.M., Fath K.R., Tsiola A., Khalpari O., Banerjee I.A. (2011). Ellagic acid promoted biomimetic synthesis of shape-controlled silver nanochains. Nanotechnology.

[98] Frayne S.H., Barnaby S.N., Nakatsuka N., Banerjee I.A. (2012). Growth and properties of CdSe nanoparticles on ellagic acid biotemplates for photodegradation applications. Mater. Express.

[99] Kim S., Liu Y., Gaber M.W., Bumgardner J.D., Haggard W.O., Yang Y. (2009). Development of chitosan-ellagic acid films as a local drug delivery system to induce apoptotic death of human melanoma cells. J. Biomed. Mater. Res. B Appl. Biomater..

[100] Shaik M.M., Kowshik M. (2019). Ellagic acid containing collagen-chitosan scaffolds as potential antioxidative bio-materials for tissue engineering applications. Int. J. Polym. Mater. Polym. Biomater..

[101] Arulmozhi V., Pandian K., Mirunalini S. (2013). Ellagic acid encapsulated chitosan nanoparticles for drug delivery system in human oral cancer cell line (KB). Colloids Surf. B Biointerfaces.

[102] Mady F.M., Shaker M.A. (2017). Enhanced anticancer activity and oral bioavailability of ellagic acid through encapsulation in biodegradable polymeric nanoparticles. Int. J. Nanomed..

[103] Vilela C., Pinto R.J.B., Coelho J., Domingues M.R.M., Daina S., Sadocco P., Santos S.A.O., Freire C.S.R. (2017). Bioactive chitosan/ellagic acid films with UV-light protection for active food packaging. Food Hydrocoll..

[104] Zhang X.-K., He F., Zhang B., Reeves M.J., Liu Y., Zhao X., Duan C.-Q. (2018). The effect of prefermentative addition of gallic acid and ellagic acid on the red wine color, copigmentation and phenolic profiles during wine aging. Food Res. Int..

[105] González-Sarrías A., García-Villalba R., Núñez-Sánchez M.Á., Tomé-Carneiro J., Zafrilla P., Mulero J., Tomás-Barberán F.A., Espín J.C. (2015). Identifying the limits for ellagic acid bioavailability: A crossover pharmacokinetic study in healthy volunteers after consumption of pomegranate extracts. J. Funct. Foods.

[106] Cerdá B., Tomás-Barberán F.A., Espín J.C. (2005). Metabolism of antioxidant and chemopreventive ellagitannins from strawberries, raspberries, walnuts, and oak-aged wine in humans: Identification of biomarkers and individual variability. J. Agric. Food Chem..

[107] Gonzalez-Sarrias A., Gimenez-Bastida J.A., Garcia-Conesa M.T., Gomez-Sanchez M.B., Garcia-Talavera N.V., Gil-Izquierdo A., Sanchez-Alvarez C., Fontana-Compiano L.O., Morga-Egea J.P., Pastor-Quirante F.A. (2010). Occurrence of urolithins, gut microbiota ellagic acid metabolites and proliferation markers expression response in the human prostate gland upon consumption of walnuts and pomegranate juice. Mol. Nutr. Food Res..

[108] Tomás-Barberán F.A., Espín J.C., García-Conesa M.T., Quideau S. (2009). Ellagitannins bioavailability and metabolism of ellagic acid and ellagitannins. Chemistry and Biology of Ellagitannins: An Underestimated Class of Bioactive Plant Polyphenols.

[109] Tomás-Barberán F.A., Gonzalez-Sarrias A., García-Villalba R., Núñez-Sánchez M.Á., Selma M.V., Garcia-Conesa M.T., Espín J.C. (2017). Urolithins, the rescue of “old” metabolites to understand a “new” concept: Metabotypes as a nexus among phenolic metabolism, microbiota dysbiosis, and host health status. Mol. Nutr. Food Res..

[110] Beshbishy A.M., Batiha G.E., Yokoyama N., Igarashi I. (2019). Ellagic acid microspheres restrict the growth of *Babesia* and *Theileria in vitro* and *Babesia microti in vivo*. Parasit. Vectors.

[111] Bulani V.D., Kothavade P.S., Nagmoti D.M., Kundaikar H.S., Degani M.S., Juvekar A.R. (2015). Characterisation and anti-inflammatory evaluation of the inclusion complex of ellagic acid with hydroxypropyl-β-cyclodextrin. J. Incl. Phenom. Macrocycl. Chem..

[112] Ratnam D.V., Chandraiah G., Meena A.K., Ramarao P., Ravi Kumar M.N.V. (2009). The co-encapsulated antioxidant nanoparticles of ellagic acid and coenzyme Q10 ameliorates hyperlipidemia in high fat diet fed rats. J. Nanosci. Nanotechnol..

[113] Ruan J., Yang Y., Yang F., Wan K., Fan D., Wang D. (2018). Novel oral administrated ellagic acid nanoparticles for enhancing oral bioavailability and anti-inflammatory efficacy. J. Drug. Deliv. Sci. Tec..

[114] Savic I.M., Jocic E., Nikolic V.D., Popsavin M.M., Rakic S.J., Savic-Gajic I.M. (2018). The effect of complexation with cyclodextrins on the antioxidant and antimicrobial activity of ellagic acid. Pharm. Dev. Technol..

[115] Wang S.-T., Chou C.-T., Su N.-W. (2017). A food-grade self-nanoemulsifying delivery system for enhancing oral bioavailability of ellagic acid. J. Funct. Foods.

[116] Selma M.V., Beltrán D., García-Villalba R., Espín J.C., Tomás-Barberán F.A. (2014). Description of urolithin production capacity from ellagic acid of two human intestinal *Gordonibacter* species. Food Funct..

[117] Selma M.V., Beltrán D., Luna M.C., Romo-Vaquero M., García-Villalba R., Mira A., Espín J.C., Tomás-Barberán F.A. (2017). Isolation of human intestinal bacteria capable of producing the bioactive metabolite Isourolithin A from ellagic acid. Front. Microbiol..

[118] Gaya P., Peirotén Á., Medina M., Álvarez I., Landete J.M. (2018). Bifidobacterium pseudocatenulatum INIA P815: The first bacterium able to produce urolithins A and B from ellagic acid. J. Funct. Foods.

[119] Fujita Y., Komagoe K., Sasaki Y., Uehara I., Okuda T., Yoshida T. (1987). Studies on inhibition mechanism of autoxidation by tannins and flavonoids. I. Inhibition mechanism of tannins on Cu (II)-catalyzed autoxidation of ascorbic acid. Yakugaku Zasshi.

[120] Kilic I., Yeşiloǧlu Y., Bayrak Y. (2014). Spectroscopic studies on the antioxidant activity of ellagic acid. Spectrochim. Acta Part A Mol. Biomol. Spectrosc..

[121] Dröge W. (2002). Free radicals in the physiological control of cell function. Physiol. Rev..

[122] Jakus V. (2000). The role of free radicals, oxidative stress and antioxidant systems in diabetic vascular disease. Bratisl. Lek. Listy.

[123] Levonen A.-L., Vahakangas E., Koponen J.K., Yla-Herttuala S. (2008). Antioxidant gene therapy for cardiovascular disease: Current status and future perspectives. Circulation.

[124] Morel Y., Barouki R. (1999). Repression of gene expression by oxidative stress. Biochem. J..

[125] Rice-Evans C., Miller N., Paganga G. (1997). Antioxidant properties of phenolic compounds. Trends Plant. Sci..

[126] Vattem D.A., Shetty K. (2005). Biological functionality of ellagic acid: A review. J. Food Biochem..

[127] Boehning A.L., Essien S.A., Underwood E.L., Dash P.K., Boehning D. (2018). Cell type-dependent effects of ellagic acid on cellular metabolism. Biomed. Pharmacother..

[128] Zeb A. (2018). Ellagic acid in suppressing *in vivo* and *in vitro* oxidative stresses. Mol. Cell. Biochem..

[129] Ríos J.L., Giner R.M., Marín M., Recio M.C. (2018). A pharmacological update of ellagic acid. Planta Med..

[130] Bialonska D., Kasimsetty S.G., Schrader K.K., Ferreira D. (2009). The effect of pomegranate (*Punica granatum* L.) byproducts and ellagitannins on the growth of human gut bacteria. J. Agric. Food Chem..

[131] Funatogawa K., Hayashi S., Shimomura H., Yoshida T., Hatano T., Ito H., Hirai Y. (2004). Antibacterial activity of hydrolyzable tannins derived from medicinal plants against *Helicobacter pylori*. Microbiol. Immunol..

[132] Asres K., Bucar F., Edelsbrunner S., Kartnig T., Höger G., Thiel W. (2001). Investigations on antimycobacterial activity of some Ethiopian medicinal plants. Phyther. Res..

[133] Kolodziej H., Kayser O., Kiderlen A., Ito H., Hatano T., Yoshida T., Foo L. (2001). Antileishmanial activity of hydrolyzable tannins and their modulatory effects on nitric oxide and tumour necrosis factor-alpha release in macrophages *in vitro*. Planta Med..

[134] Soh P.N., Witkowski B., Olagnier D., Nicolau M.L., Garcia-Alvarez M.C., Berry A., Benoit-Vical F. (2009). *In vitro* and *in vivo* properties of ellagic acid in malaria treatment. Antimicrob. Agents Chemother..

[135] Ascacio-Valdés J., Burboa E., Aguilera-Carbo A.F., Aparicio M., Pérez-Schmidt R., Rodríguez R., Aguilar C.N. (2013). Antifungal ellagitannin isolated from *Euphorbia antisyphilitica* Zucc. Asian Pac. J. Trop. Biomed..

[136] Vilhelmova-Ilieva N., Jacquet R., Quideau S., Galabov A.S. (2014). Ellagitannins as synergists of ACV on the replication of ACV-resistant strains of HSV 1 and 2. Antiviral Res..

[137] BenSaad L.A., Kim K.H., Quah C.C., Kim W.R., Shahimi M. (2017). Anti-inflammatory potential of ellagic acid, gallic acid and punicalagin A&B isolated from *Punica granatum*. BMC Complement. Altern. Med..

[138] Akileshwari C., Raghu G., Muthenna P., Mueller N.H., Suryanaryana P., Petrash J.M., Reddy G.B. (2014). Bioflavonoid ellagic acid inhibits aldose reductase: Implications for prevention of diabetic complications. J. Funct. Foods.

[139] Al-Sayed E., El-Naga R.N. (2015). Protective role of ellagitannins from *Eucalyptus citriodora* against ethanol-induced gastric ulcer in rats: Impact on oxidative stress, inflammation and calcitonin-gene related peptide. Phytomedicine.

[140] Kannan M.M., Quine S.D. (2013). Ellagic acid inhibits cardiac arrhythmias, hypertrophy and hyperlipidaemia during myocardial infarction in rats. Metabolism.

[141] Rogerio A.P., Fontanari C., Borducchi É., Keller A.C., Russo M., Soares E.G., Albuquerque D.A., Faccioli L.H. (2008). Anti-inflammatory effects of *Lafoensia pacari* and ellagic acid in a murine model of asthma. Eur. J. Pharmacol..

[142] Ortiz-Ruiz C.V., Berna J., Tudela J., Varon R., Garcia-Canovas F. (2016). Action of ellagic acid on the melanin biosynthesis pathway. J. Dermatol. Sci..

